# ENDOXY - Development of a Biomimetic Oxygenator-Test-Device

**DOI:** 10.1371/journal.pone.0142961

**Published:** 2015-12-18

**Authors:** Maren Dietrich, Nicole Finocchiaro, Sebastian Olszweski, Jutta Arens, Thomas Schmitz-Rode, Joerg Sachweh, Stefan Jockenhoevel, Christian G. Cornelissen

**Affiliations:** 1 Department of Tissue Engineering & Textile Implants, Institute of Applied Medical Engineering, Helmholtz Institute Aachen, RWTH Aachen University, Aachen, Germany; 2 Department of Cardiovascular Engineering, Institute of Applied Medical Engineering, Helmholtz Institute Aachen, RWTH Aachen University, Aachen, Germany; 3 Department of Internal Medicine – Section for Pneumology, RWTH Aachen University Hospital, Aachen, Germany; Politecnico di Milano, ITALY

## Abstract

**Objective:**

This study focusses on the development of a biomimetic oxygenator test device. Due to limited biocompatibility, current oxygenators do not allow mid- to long-term therapy. Tissue engineering uses autologous cell sources to overcome the immunogenic barriers of biomaterials. Surface coating with endothelial cells might improve hemocompatibility and thus prevent immunogenic reactions of the body. In this study this concept is applied to endothelialise a gas-permeable membrane to develop a biomimetic oxygenator test-device (ENDOXY).

**Methods:**

ENDOXY—a multifunctional test-system was developed to endothelialise a gas-permeable membrane suitable for cell culture and to test the cell retention under shear stress and to measure gas transfer through it.

**Results:**

Successful endothelialisation of the membrane was achieved and cells showed characteristic endothelial morphologies. They stained positive for endothelial markers. The number of cells aligned with shear stress and cell retention after blood perfusing experiments was high. Gas transfer is observed via uncoated and endothelialised membranes.

**Conclusion:**

The study showed promising results with regard to system design, endothelialisation, and cell retention under shear stress conditions. It strongly encourages further research into the system by testing different membrane materials to design a biomimetic membrane surface and pave way for a fully hemocompatible oxygenator.

## Introduction

Patients suffering from terminal lung failure are treated with mechanical ventilation and, if necessary, extracorporeal membrane oxygenation (ECMO) while waiting for recovery or a donor lung. ECMO, as a maximally invasive means of lung assist, is accompanied by limited hemocompatibility, an activation of the coagulation system and the complement system, plasma leakage, and protein deposition leading to a decrease in the gas exchange performance [1]. In current lung assist systems the tubing, cannulas, and oxygenator have a non-vital coating with heparin to increase the hemocompatibility and to reduce the need for systemic anticoagulation. The limitations of current oxygenator devices have constrained the mid to long-term use of these devices and the development of an implantable lung assist system. To overcome these limitations and risks, a vital and functional coating may be a solution.

Initiated by tissue engineering and regenerative medicine, endothelial cell coating is used in a variety of applications to improve hemocompatibility [2] of medical devices and implants. In cardiovascular implants such as stents and heart valves, endothelialisation has already been successfully performed [3, 4]. These findings encouraged us to vitalise the surface of oxygenators in lung assist systems with a functional endothelial confluent monolayer. Few other groups deal with the topic of coating endothelial cells on gaspermeable membranes: Polk et al. [5] endothelialised modified microporous hollow fibres for a biohybrid artificial lung prototype and Hess and co-workers [6] endothelialised poly 4-methyl-1-pentene (PMP) gas exchange membranes under static culture conditions.

In our present study flat membrane sheets were introduced in the newly developed multifunctional *in-vitro* test system—ENDOXY. This system allows testing the endothelialisation of gas permeable membranes under shear stress and the gas transfer.

In the vasculature, the endothelium is responsible for the hemocompatibility [6–9]. Fluid shear stress on endothelial cells plays an important role within the vascular system. Endothelial cells (EC) alter their morphology, function, and gene expression in response to shear stress [10]. It was demonstrated that unidirectional flow leads to elongated spindle-shaped ECs and corresponds to the healthy, physiological phenotype whereas statically cultured or disturbed flow ECs show the typical cobblestone morphology with no uniform direction which is more of the thrombogenic and inflammatory phenotype [10, 11]. Moreover fluid shear stress on ECs induces the expression of antithrombotic and anti-inflammatory components and cytokines into the blood stream [10]. To benefit from the advantages of ECs biomedical implants, e.g. vascular grafts, were coated with ECs by static cultivation followed by dynamical preconditioning [4, 12]. It was shown that preconditioning of ECs for shear stress leads to significant higher cell retention.

Taking all this into account, the idea of this study is the translation of a viable biomimetic endothelial interface onto a gas permeable membrane. To realise this idea we developed a multifunctional *in-vitro* flow chamber system—the ENDOXY-test-system (i) with integrated flat membrane sheets that were endothelialised, conditioned for shear stress (ii), exposed to blood statically (iii) as well as dynamically, including the measurement of gas transfer through the cell-free and cell-seeded membrane (iv).

## Materials and Methods

### Development of the ENDOXY-Test-System and System Set-up

**Fig 1:**
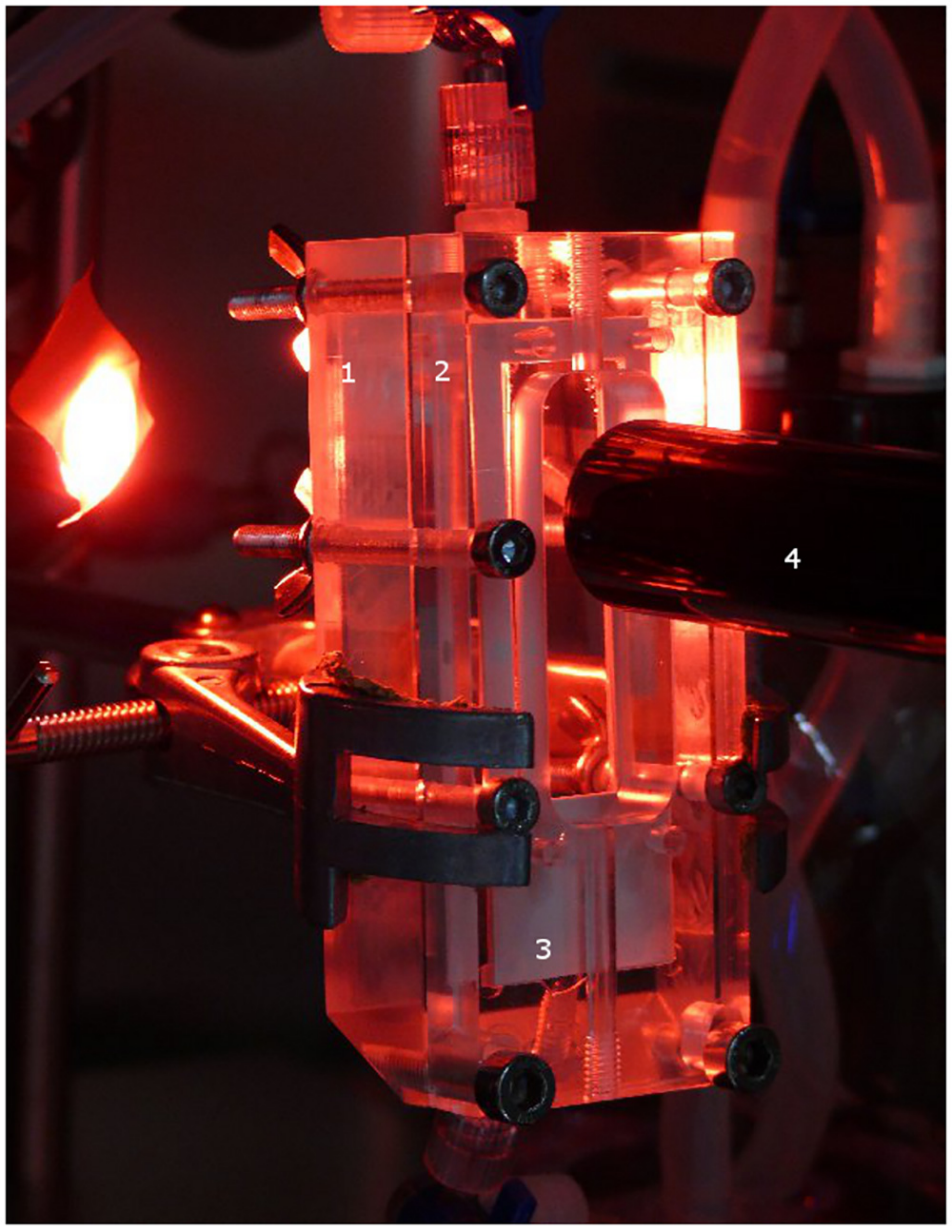
The ENDOXY system set up as a bioreactor for application of defined shear stress on endothelial cells. 1 –gas chamber, 2 –medium / blood chamber, 3 –Lumox^™^ slide, 4 –microscope.

The test-system is a multifunctional device, which can be used as a bioreactor and oxygenator device (Fig 1). Cells can be cultured and conditioned directly in the system and cell growth is controlled online by a microscopic camera (T.I.M.M, SPI GmbH, Oppenheim, Germany). Ultra-thin (25μm) gas-permeable Lumox^™^-Slides (Sarstedt, Nümbrecht; Germany) were used as membrane. This membrane is suitable for cell culture.

**Fig 2:**
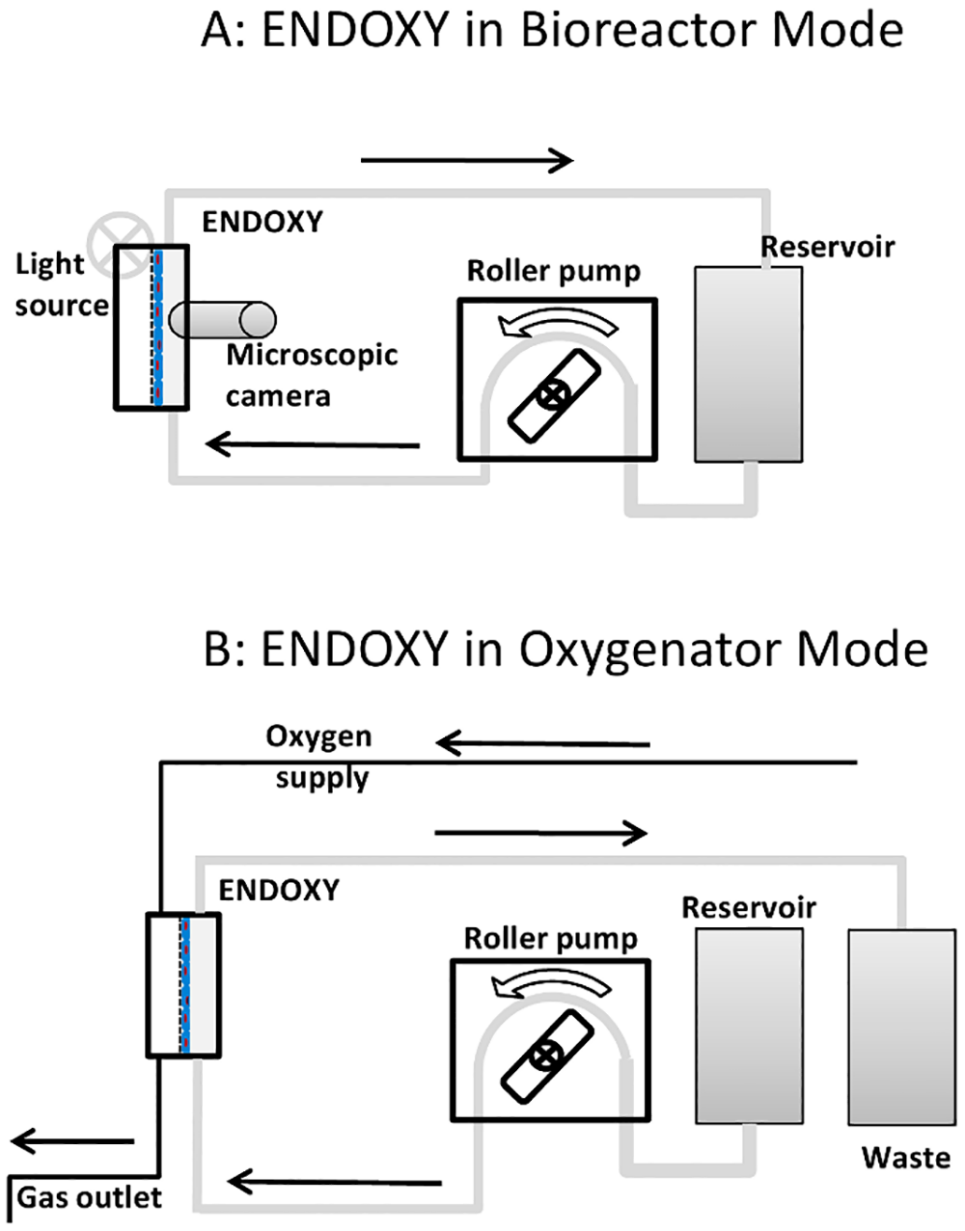
Schematic drawing of the ENDOXY system set up in bioreactor mode (A) and oxygenator mode (B).

The bioreactor itself consists of one medium/blood chamber and one oxygen chamber. One lumox^™^-Slide (Fig 1, white) is fixed between the two chambers with cells in direction of the medium/blood chamber and sealed with a silicon membrane. The system (Fig 2A) is mounted together and via silicon tubes (Tygon SI, Ismatec,) the device is connected to a medium reservoir. The medium is transported through the system by a roller pump (ISMATEC, MCP Process ISM 915). The whole system is placed in an incubator at 37°C and 5% CO_2_. When the system is switched to oxygenation (Fig 2B) the cell culture medium is replaced by blood and connected to the oxygen supply for gas transfer. The blood and oxygen flow run in counter-current.

The width of the ENDOXY system is 18 mm, defined by the Lumox^™^ membrane’s width. The perfusion chamber is 0.8 mm in height. The membrane’s surface area is 9 cm^2^. Shear stress on endothelial cells can thus be calculated for cell culture medium by:

$$\tau =\frac{6\;\times Viscosity\;\times Flow\;rate}{Width\;\times Height^{2}}=\frac{6\;\times 0.0014\;Pa\times s\times \;Flow\;rate}{18\;mm\times 0.8^{2}\times mm^{2}}$$

### Cell culture

Primary HUVECs (human umbilical vein endothelial cells) were isolated from human umbilical cord provided by Department of Gynaecology and Obstetrics of the University Hospital Aachen in accordance with the human subjects approval of the local ethics committee of the RWTH Aachen University Hospital (votum #EK 2067) after obtaining written consent. The approval includes the use of umbilical cord-derived cells for *in vitro* studies in tissue engineering. The umbilical cord was washed with 37°C Phosphate Buffered Saline (PBS) and was cannulated. 2.4 IU/mL Dispase-solution (Roche^®^) was filled into the vein. The vein was incubated for 30 min at 37°C and 5% CO_2_. Detached HUVECs were flushed with 37°C pre-warmed Phosphate Buffer Saline (PBS) into a 50 mL falcon tube (Falcon BD) and centrifuged at 500 g for 5 min. The supernatant was discarded and the cell pellet was resuspended in EBM-2 (Lonza^®^) supplemented with EGM-2 SingleQuot Kit Suppl. & Growth Factors (Lonza^®^) and 1% antibiotic/antimycotic solution (Gibco^®^). The resuspended cells were plated into cell culture flasks (75 cm^3^, Greiner bio-one), pre-coated with gelatine (Gelatine Type B, Sigma) and were maintained in a humidified incubator at 37°C and 5% CO_2_ and cultivated to 80% confluency. For all experiments, pooled cells from four different donors were used in passage four.

#### Endothelialisation of Lumox^™^-membrane

1*10^5^ HUVECs per mL medium (20000–30000 cells /cm^2^) are cultivated on one Lumox^™^ slide. A total of 2–3 mL EBM-2 supplemented with EGM-2 SingleQuot was added per slide. Cultivation takes place in a humidified incubator at 37°C and 5% CO_2_. Medium is changed every second day. Cell growth and shape were observed by a light microscope (AxioImager; Carl Zeiss, Germany).

For dynamic cultivation of HUVECs, cells were coated on Lumox^™^ -SlideFlasks for 3–4 days as described before and then the slide was transferred into the ENDOXY-System. The system was filled with 200 mL EBM-2 plus supplements. Cell growth was controlled online by a microscopic camera (T.I.M.M, SPI GmbH, Oppenheim; Germany) and cells were cultivated until confluence. Table 1 shows flow rates, which were increased for 3 consecutive days and corresponding shear stress in the perfused system. Experiments were conducted in triplicate.

**Table 1:** Flow rates generated in the ENDOXY during dynamic cell culture.

Day of dynamic culture	Flow rate (ml/min)	Shear stress (dyn/cm²)
1	20	2.5
2	30	3.7
3	40	4.9

#### Incubation and perfusion of HUVECs with blood

For static incubation of HUVECs with blood, the cells were cultivated under standard conditions in Lumox^™^ slide flasks (Greiner Bio-One, Germany) until confluence. Then cell culture medium was removed and the cells were incubated with blood (2 mL) for 90 min. After the incubation the slides were washed with pre-warmed EBM-2 (37°C) and fluorescence stained. For the dynamic incubation with blood, HUVECs were cultivated till confluence in the ENDOXY-system under dynamic conditions as delineated above. During oxygenator experiments (see next sections) the cells were perfused with blood for 180 min. Before analysis, the slides were washed with pre-warmed EBM-2 (37°C) and were fluorescence stained. Control cells were incubated with EBM-2 cell culture medium. All blood incubation experiments were carried out in triplicate.

### Analysis of endothelialised Lumox^™^-Membrane

#### Conventional light microscopy

For light microscopic examination for general cell growth, slides were analysed using routine bright field contrast enhanced light microscopy (AxioImager; Carl Zeiss, Germany) without staining. Images were acquired using a high-resolution CCD colour camera (AxioCam MRc; Carl Zeiss, Germany).

#### Immunohistochemistry

Immunohistochemical characterization of samples was performed directly on the Lumox^™^-slides. Cells were fixed in methanol for 10 min at -20°C and rehydrated in PBS for 10 min. Samples were blocked with 3% BSA (Bovine Serum Albumin) 30 min. Slides were incubated for 1 h at 37°C with mouse monoclonal anti-CD31 (1:100; Sigma) and rabbit polyclonal anti-vWF (1:100; DAKO, Glostrup, Denmark). The slides were then incubated for 1 h at room temperature with either Alexa Fluor^®^594 (1:100; A11020, Invitrogen) or Alexa Fluor^®^488 (1:100; A24922, Invitrogen) secondary antibodies. Cell nuclei were counterstained using Roti^®^-Mount FluorCare DAPI (Roth, Cat. No. HP20.1). For negative controls, samples were incubated in diluents and the secondary antibody. Samples were viewed using a fluorescence microscope (AxioObserver Z1; Carl Zeiss GmbH), and images were acquired using a monochrome camera (AxioCam MRm; Carl Zeiss GmbH, Germany).

### Gas transfer measurement

Gas transfer measurements were conducted according to standardized protocols defined in DIN 12022/ ISO 7199. Gas transfer measurements were performed with cell-free and cell-populated Lumox^™^-slides. When using cell-free slides the system assembly was washed with physiological sodium chloride solution before filling the system with blood. Slides coated with cells were grown in the ENODXY-test-system and for gas transfer measurements cell culture medium was replaced by blood. The system was perfused with twice of its filling volume with blood before starting. Samples were taken in triplicate before oxygenation and after oxygenation and were analyzed in a blood gas analyzer (AB2 800 Flex, Radiometer GmbH, Willich, Germany). The partial pressure of oxygen pO_2_ and carbon dioxide pCO_2_, oxygen saturation sO_2_ and hemoglobin content Hb were registered. The sample collection was performed at different flow rates (10 mL/min, 5 mL/min, 2.5 mL/min), which are comparable to flow rates in conventional oxygenators. The surface area of a MEDOS hilite^™^ 7000 LT oxygenator, a typical oxygenator for ECMO, is 1.9 m^2^. This oxygenator is designed for flow rates up to 7 L/min, which, comparing the differences in surface, translates to 3.3 mL/min in the ENDOXY system. Gas transfer measurements were performed on one uncoated and one cell coated membrane. According to DIN 12022/ ISO 7199, oxygen flow was set to ¼ of the blood perfusion rate.

For calculation of the oxygen gas transfer, mean values for pO_2_, sO_2_ and Hemoglobin were generated for each flow rate at each of the two sampling points. Oxygen content was calculated for each sampling point by the following formula:

$$\left(Hb\;\times Oxygen\;saturation\;\times 1.36\;\frac{ml}{g}\right)+\;\left(Oxygen\;partial\;pressure\;\times 0.0031\;\frac{ml}{mmHg}\right).$$

The difference in oxygen content before and after the ENDOXY system was calculated. Subsequently, the result was adjusted for the flow rate and the area of gas transfer.

Maximum theoretical oxygen transfer through uncoated Lumox^™^ membranes was calculated from PTFE’s gas transfer coefficient for oxygen[13]:

$$5\times 10^{-12}\;\times \;\frac{ml}{sec\times cm\times mmHg}.$$

For the calculation of the theoretical maximum of oxygen gas transfer through endothelialized membranes, the endothelial layer’s thickness was estimated to be 5 μm [14]. The oxygen gas transfer coefficient of tissue is [15]:

$$2.5\times 10^{-12}\;\times \;\frac{ml}{sec\times cm\times mmHg}$$

Thus, expected theoretical gas transfer through the cell—membrane system could be calculated by the following formula:

$$\frac{760\;mmHg\;-\;pO_{2\;inlet\;ENDOXY}}{d_{Lumox}+\;\left(2\;\times \;d_{Zellen}\right)}\times 5\times 10^{-12}\;\times \;\frac{ml\;\times \;cm}{sec\times cm^{2}\times mmHg}.$$

### Redesign of the ENDOXY system & Gas transfer measurements

After gas transfer measurements were conducted in the original ENDOXY system, it was extensively modified to allow for gas transfer measurements on endothelialized membranes. The thickness of the blood film affects the gas transfer as it defines the maximum of the diffusion distance; accordingly, it was reduced from 800 μm to 300 μm. The second major parameter that can be easily tailored is the time of blood contact for a given flow. By doubling the length of the system from 5 cm to 10 cm the contact time was also doubled; this lead to an increase in surface area to 12 cm^2^. Blood perfusion flow rates tested in the system ranged from 1 to 3 ml/min. In all other aspects, testing was performed as already described.

### Statistical analysis

Statistical analysis was performed on the results of gas transfer measurement. Continuous variables are expressed as mean ± standard deviation. The difference in oxygen saturation between the inlet and the outlet of the ENDOXY was assessed by a student T-Test after assessment of normal distribution. All statistical tests were 2-tailed and a p-value < 0.05 was considered statistically significant. Data analysis was performed using commercially available software (Microsoft Office Excel, The Microsoft Corporation, USA & SAS enterprise guide version 4, SAS Institute Inc., USA).

## Results

### Comparison of static and dynamic cultivated HUVECs in cell culture medium on Lumox^™^ -Membranes

**Fig 3:**
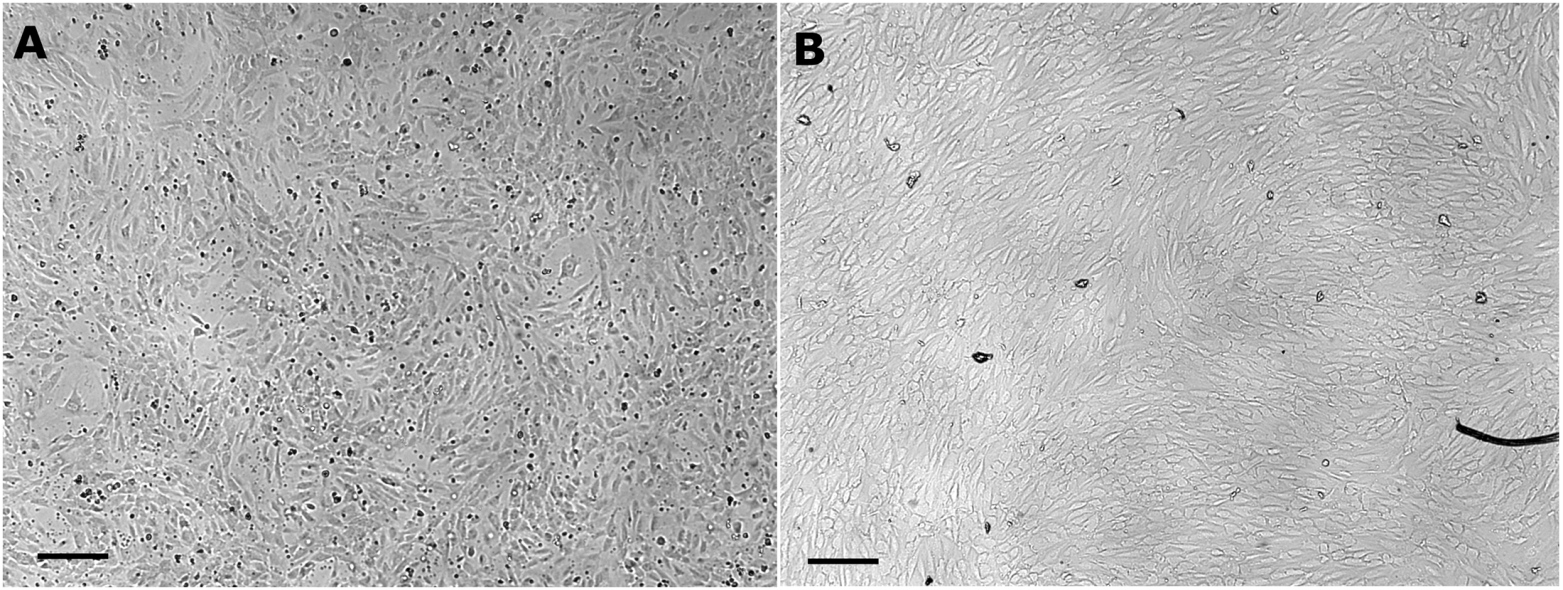
Comparative light-microscopic picture of HUVECs cultured on Lumox^™^ slides for 7 days. A –Static control. B—Shear stress stimulated cells. Scale bar = 200 μm.

**Fig 4:**
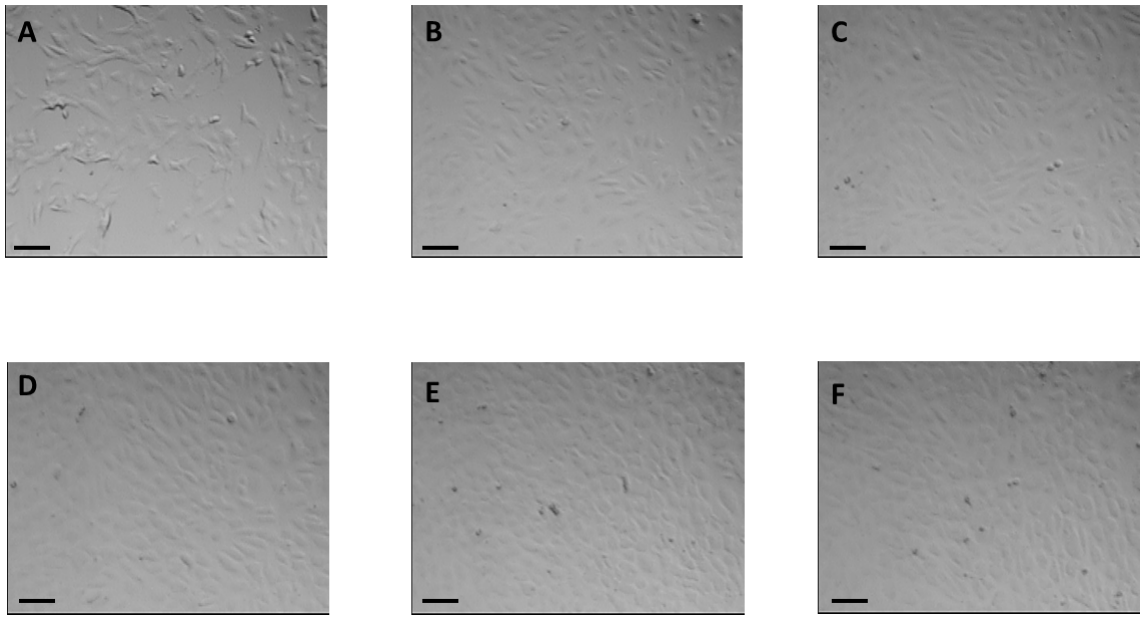
On-line microscopy of endothelial cells cultured in the ENDOXY system during the first 36 hours. Scale bar = 100 μm.

Both culture conditions, static and dynamic, lead to a confluent cell layer (Fig 3). Cells grown under static conditions show the typical cobblestone shape and dynamically cultivated cells show an elongated flat shape (Figs 3 and 4). On-line microscopy (Fig 4) reveals an increase in cell number predominantly during the first 12 hours of dynamic culture (t_0h_ = 319 ± 22 cells / mm^2^, t_12h_ = 503 ± 17 cells / mm^2^, t_36h_ = 551 ± 39 cells / mm^2^). At the start of the dynamic culture process (Fig 4A), cells exhibit the “cobblestone” morphology of endothelium in culture. Under shear stress, cells develop an elongated morphology and progressively align along a common vector (Fig 4B to 4F).

**Fig 5:**
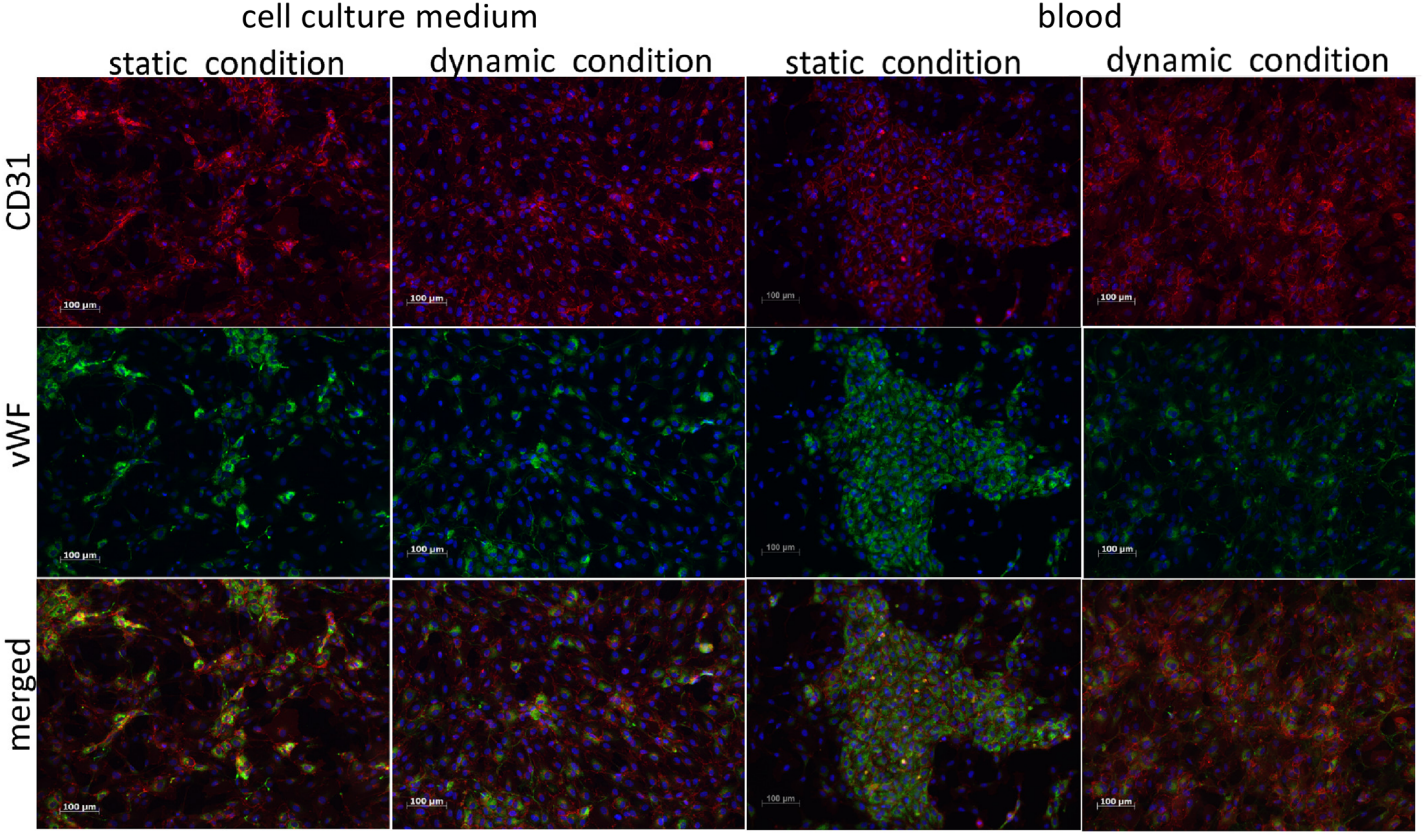
Fluorescence staining against CD31 and vWF for dynamically and statically cultured endothelial cells incubated with either cell culture medium or blood. Static incubation incubation with blood was done for 90 minutes whereas blood was perfused for 180 minutes. All samples stain positive for CD31 and vWF, proving endothelial lineage. Blood incubation does not alter marker expression. Scale bar = 100 μm.

Independently of culture conditions all samples stain positive for CD31 and vWF (Fig 5). The typical subcellular localisation of the different markers can be observed: CD31 is localized on the membrane (red stain) at points of cell-cell contact and vWF is located in the cytoplasma (green stain). The merged stains give evidence of the cell shape and alignment. Noticeable is the non-uniform distribution of the markers in the static cultured cells (right rows) in contrast to the dynamically cultivated cells (left rows). They appear more evenly distributed and aligned with the flow. The fluorescence signal for CD31 is uniformly distributed. The signal for vWF in the dynamically cultivated cells is less intense and less uniformly distributed than in statically cultured cells. In the contrary to the dynamic cultured cells the static cultured cells show hotspots of both markers, identified by the stronger fluorescence signal.

### Analysis of Cell-Coated Lumox^™^-Membranes Exposed to Blood

In comparison to the samples exposed to medium no difference in marker expression can be observed if exposed to blood (Fig 5). Cell retention after static and dynamic blood incubation compared to cell retention after dynamic and static medium incubation is the same.

### Gas transfer measurement

Blood gas analysis data from gas transfer tests on uncoated and coated Lumox membranes^™^ is given in Tables 2 and 3. Gas transfer testing with whole blood led to low differences in oxygen saturation and partial pressure before and after the ENDOXY system due to absorption of the O_2_ molecules by haemoglobin. Thus, only two gas transfer measurements on uncoated membranes delivered statistically significant differences in oxygen saturation, warranting further analysis (Table 4). The maximum gas transfer rate measured on uncoated membranes was $8.50\;\pm 6.85\;\times 10^{-5}\frac{ml}{sec\times {cm}^{2}}$. The predicted maximum gas transfer through uncoated membranes is $14.11\;\times \;10^{-5}\frac{ml}{sec\times {cm}^{2}}$. Differences in oxygen saturation before and after the ENDOXY system after cell coating were not significant. Thus, no gas transfer rate could be calculated. The predicted maximum gas transfer through coated membranes is $8\;\times 10^{-5}\;\frac{ml}{sec\times {cm}^{2}}$.

**Table 2:** Gas transfer measurement of uncoated Lumox^™^ slide—blood gas analysis.

Flow rate	Pre / Post ENDOXY	pO_2_ (mmHg)	sO_2_ (%)		pCO_2_ (mmHg)	Hemoglobin (g/dl)
10 ml / min	Pre	54.6 ± 0.8	68.6 ± 0.7	p = 0.03	53.2 ± 1.0	10.8 ± 0.5
	Post	57.5 ± 1.8	72.8 ± 2.0		52.2 ± 1.3	10.6 ± 0.2
7.5 ml / min	Pre	68.6 ± 26.2	66.1 ± 1.0	n.s.	33.1 ± 16.0	14.2 ± 0.1
	Post	53.2 ± 0.6	67.9 ± 1.2		51.6 ± 0.6	13.9 ± 1.0
5 ml / min	Pre	54.6 ± 0.2	68.1 ± 0.3	n.s.	50.1 ± 1.0	13.5 ± 0.8
	Post	57.5 ± 1.7	70.6 ± 1.6		33.0 ± 14.9	12.6 ± 1.0
2.5 ml / min	Pre	53.2 ± 0.2	68.2 ± 0.3	p = 0.02	48.0 ± 0.5	18.1 ± 0.4
	Post	54.2 ± 1.2	70.5 ± 1.0		47.7 ± 0.3	17.8 ± 0.4

Blood gas analysis on blood samples acquired from the gas transfer test loop at the ENDOXY’s inlet (Pre) and outlet (Post). Based on results of the T-Test on oxygen saturation values, gas transfer calculation was performed for flow rates of 10 ml / min and 2.5 ml / min. n.s. = not significant.

**Table 3:** Gas transfer measurement of cell coated Lumox^™^ slide—blood gas analysis.

Flow rate	Pre / Post ENDOXY	pO_2_ (mmHg)	sO_2_ (%)		pCO_2_ (mmHg)	Hemoglobin (g/dl)
10 ml / min	Pre	41.0 ± 0.3	58.3 ± 1.4	n.s.	52.4 ± 3.8	12.8 ± 1.0
	Post	41.2 ± 0.2	59.9 ± 0.3		47.8 ± 0.1	11.7 ± 0.1
5 ml / min	Pre	40.8 ± 0.5	57.9 ± 0.3	n.s.	50.4 ± 4.6	12.5 ± 0.9
	Post	40.8 ± 1.0	57.8 ± 0.8		51.4 ± 2.3	12.7 ± 1.4
2.5 ml / min	Pre	40.8 ± 0.6	58.4 ± 0.6	n.s.	50.9 ± 0.3	12.1 ± 0.3
	Post	41.2 ± 0.9	59.8 ± 1.4		50.3 ± 0.3	12.1 ± 0.1

Blood gas analysis on blood samples acquired from the gas transfer test loop at the ENDOXY’s inlet (Pre) and outlet (Post). The difference of oxygen saturation at the inlet and outlet is statistically not significant, prohibiting further analysis. n.s. = not significant.

**Table 4:** Gas transfer of uncoated Lumox^™^ slide.

Flow rate	Measured gas transfer $\left(\frac{ml}{\text{sec}\;\times \;cm²}\right)$	Maximum of gas transfer $\left(\frac{ml}{\text{sec}\;\times \;cm²}\right)$
10 ml / min	8.50 × 10^−5^ ± 6.85 × 10^−5^	14.11 x 10^−5^
2.5 ml / min	1.21 × 10^−5^ ± 2.04 x 10^−5^	14.11 x 10^−5^

Calculated gas transfer through uncoated Lumox^™^ slides. Results show high standard deviations. Still, the measured gas transfer at 10 ml / min blood flow demonstrates an efficient system setup as the measured transfer is near the theoretical maximum.

### Gas transfer measurement after redesign of the ENDOXY system

Blood gas analysis data from gas transfer tests on uncoated Lumox membranes^™^ after redesign of the ENDOXY system is given in Table 5. Differences in oxygen saturation and partial pressure before and after the system using whole blood were well detectable. The maximum gas transfer (Table 6) through uncoated membranes was $9.1\;\pm 1.2\;\times \;10^{-5}\;\frac{ml}{sec\times {cm}^{2}}$, indicating a progress in system design.

**Table 5:** Gas transfer measurement of uncoated Lumox^™^ slide after redesign—blood gas analysis.

Flow rate	Pre / Post ENDOXY	pO_2_ (mmHg)	sO_2_ (%)		pCO_2_ (mmHg)	Hemoglobin (g/dl)
3 ml / min	Pre	40.7 ± 0.7	69.5 ± 1.2	p < 0.05	46.9 ± 0.7	11.2 ± 0.2
	Post	59.7 ± 5.1	87.7 ± 1.2		47.1 ± 0.6	13.9 ± 0.3
2 ml / min	Pre	42.1 ± 0.3	65.1 ± 0.7	p < 0.05	49.7 ± 0.2	10.7 ± 0.1
	Post	53.9 ± 3.7	85.4 ± 2.5		49.2 ± 0.6	13.9 ± 0.4
1 ml / min	Pre	41.9 ± 0.9	62.1 ± 2.4	p < 0.05	50.3 ± 0.8	10.0 ± 0.4
	Post	53.1 ± 1.3	91.4 ± 2.4		50.4 ± 0.5	11.8 ± 0.4

Blood gas analysis on blood samples acquired from the gas transfer test loop at the ENDOXY’s inlet (Pre) and outlet (Post). Based on results of the T-Test on oxygen saturation values, gas transfer calculation was performed for all flow rates.

**Table 6:** Gas transfer of uncoated Lumox^™^ slide after redesign.

Flow rate	Measured gas transfer $\left(\frac{ml}{\text{sec}\;\;\times \;cm²}\right)$	Maximum of gas transfer $\left(\frac{ml}{\text{sec}\;\;\times \;cm²}\right)$
3 ml / min	7.27 x 10^−5^ ± 2.4 x 10^−5^	14.11 × 10^−5^
2 ml / min	9.11 x 10^−5^ ± 1.2 x 10^−5^	14.11 × 10^−5^
1 ml / min	6.28 x 10^−5^ ± 5.6 x 10^−5^	14.11 × 10^−5^

Calculated gas transfer through uncoated Lumox^™^ slides, measured in the redesigned ENDOXY system.

Blood gas analysis data from gas transfer tests on coated Lumox membranes^™^ after redesign of the ENDOXY system is given in Table 7 with differences in oxygen saturation and partial pressure before and after the system using whole blood also being well detectable. The maximum gas transfer rate measured on coated membranes (Table 8) after redesign of the system was $8.46\;\pm 1.62\;\times \;10^{-5}\;\frac{ml}{sec\times {cm}^{2}}$.

**Table 7:** Gas transfer measurement of coated Lumox^™^ slide after redesign—blood gas analysis.

Flow rate	Pre / Post ENDOXY	pO_2_ (mmHg)	sO_2_ (%)		pCO_2_ (mmHg)	Hemoglobin (g/dl)
3 ml / min	Pre	37.0 ± 0.7	52.4 ± 0.9	p < 0.05	50.7 ± 1.8	11.9 ± 0.7
	Post	39.7 ± 1.1	58.7 ± 2.4		48.0 ± 2.6	10.9 ± 1.0
2 ml / min	Pre	35.1 ± 0.2	46.5 ± 0.6	p < 0.05	55.8 ± 0.4	11.8 ± 0.1
	Post	41.6 ± 1.4	60.6 ± 3.4		55.9 ± 0.9	12.8 ± 0.1
1 ml / min	Pre	35.6 ± 0.1	49.5 ± 0.6	p < 0.05	52.5 ± 0.7	11.4 ± 0.2
	Post	50.6 ± 2.2	74.7 ± 2.1		52.3 ± 0.5	13.0 ± 0.4

Blood gas analysis on blood samples acquired from the gas transfer test loop at the ENDOXY’s inlet (Pre) and outlet (Post). Based on results of the T-Test on oxygen saturation values, gas transfer calculation was performed for all flow rates.

**Table 8:** Gas transfer of coated Lumox^™^ slide after redesign.

Flow rate	Measured gas transfer $\left(\frac{ml}{\text{sec}\;\times \;cm²}\right)$	Maximum of gas transfer $\left(\frac{ml}{\text{sec}\;\times \;cm²}\right)$
3 ml / min	9.54 × 10^−6^ ± 4.08 x 10^−5^	14.11 x 10^−5^
2 ml / min	8.46 × 10^−5^ ± 1.62 x 10^−5^	14.11 x 10^−5^
1 ml / min	7.64 × 10^−5^ ± 8.18 x 10^−6^	14.11 x 10^−5^

Calculated gas transfer through coated Lumox^™^ slides, measured in the redesigned ENDOXY system. Results show a high standard deviation for a blood flow of 3 ml / min. At a blood flow of 1 ml / min and 2 ml / min, gas transfer through the cell-coated membrane is detectable.

## Discussion

Mimicking nature is considered to be the ideal interface for blood contacting surfaces of implants [2, 6]. Thus, many approaches were made to endothelialise artificial surfaces of vascular grafts, heart valves, and stents to improve hemocompatibility [4, 6, 16, 17]. Due to the complex demands much less effort was put into the idea to endothelialise gas-permeable membranes for the use in oxygenators to design a biohybrid lung.

To progress the development towards a biohybrid lung we focussed on a system to test an endothelialised gas-permeable membrane with regard to the influence of shear stress and blood on the confluent vital endothelial cell layer as well as on its impact on the gas exchange performance. Existing oxygenator test procedures are designed for the evaluation of complete oxygenator modules. They do predict real-life gas transfer in oxygenators adequately, but all currently available oxygenators rely on hollow-fibre membranes unsuitable to our purposes. Gas transfer through permeable flat membranes in contrast is usually measured in an artificial test environment by applying a mixture of gases at a fixed pressure against a lower pressure at the other side of the membrane. This test setup is not comparable to the situation in oxygenator devices. Thus, we decided to develop the ENDOXY system that harbours a flat membrane and can be introduced in a standard oxygenator test loop while respecting the requirements of controlled cell culture.

The presented online imaging system allowed for the observation of cell growth, cell alignment, and confluence. Flat membrane sheets were integrated instead of capillary membranes used by others, because flat membranes have essential advantages [5, 6, 18]: the flat membrane is easy to handle, the cell seeding is convenient, efficient, and controllable. In contrast to hollow fibre membranes there is no friction between the membrane sheets that may lead to disruption of the confluent endothelial cell layer.

By applying this technique ECs did attach and formed a confluent monolayer on the Lumox^™^-slides without any additional surface treatment. In standard endothelial cell culture the surfaces of the culture dishes are coated with gelatine or fibronectin for attachment of ECs, but it has also been reported that the coatings are not essential if the medium and growth factor combination is appropriate [19–21].

The morphology of the differently cultivated ECs is distinct. Histology showed the typical cobblestone shape for statically cultivated cells and an elongated shape for dynamically cultivated cells. The change in morphology started 30 min to 1 h after applying fluid shear stress to the cells. Similar observations were reported by Michielis [7]. Ives *et al*. [22] reported that cells align with the direction of the fluid shear stress and that the alignment is influenced by the state of confluence. Our experiments also showed that less confluent cell portions aligned faster than highly confluent cells.

Cells conditioned for shear stress expressed CD31 at sights of cell-to-cell contact in a uniform way. CD31 is typically expressed at sights of endothelial intercellular junctions [23–25]. Thus it might be inferred that fluid shear stress maintains an equal and uniform expression of CD31. It is known that CD31 is mechanosensitive, i.e. it is responding to fluid shear stress [26, 27].

In confluent unactivated normal ECs vWF is present in the cell; vWF is stored in the Weible-Palade-bodies [24, 28]. It is only secreted in case of activation of the cells [2, 29]. The immunohistological staining gives evidence that the endothelial cells are in an unactivated state, because the positive signal for vWF is located in the cytoplasma.

In a second step endothelialised Lumox^™^-Membranes were exposed to or perfused with blood. Few publications report on the use of whole blood and dynamic experimental setups to test biomaterials and cells which are then used for the coating [2]. HUVECs exposed to blood did not show any detachment or change in morphology and did express markers compared to control cells. The immunohistological staining showed the same fluorescence pattern as control cells. *In vivo* the endothelium affects amongst others thrombogenicity and fibrinolysis by secretion of various substances. vWF enables platelets to attach to exposed ECM components and stabilizes factor VIII in the coagulation pathway [30]. The exposure of a confluent viable monolayer of HUVECs to blood did not have any negative effect on the cells in comparison to control cells, concerning the expression of vWF and CD31 as well as on cell morphology and cell retention.

In the final step the gas transfer through the populated and unpopulated Lumox^™^-Membranes was evaluated. Gas transfer measurement through cell-free membranes was comparable to the predicted theoretical gas transfer for a flow rate of 10 ml / min through the ENDOXY system (Table 4). This result underlines the suitability of the developed system to evaluate new flat membrane oxygenator concepts.

It was assumed that the coating with HUVECs would decrease the gas transfer through the membrane in comparison to uncoated slides by cause of increased wall thickness of the layer. The natural blood gas barrier (BGB) has a thickness ranging from 0.2 μm to 1 μm [31]. The Lumox^™^-slide-membrane by itself has a thickness of 25 μm plus the HUVEC layer, which was estimated with 5 μm [14]. This is a multiple of the thickness of the natural BGB. That decreases the diffusion, because the diffusion resistance is proportional to the thickness of the layer [28]. Still, the calculated theoretical maximum of gas transfer through the cell coated membrane—$8\;\times 10^{-5}\;\frac{ml}{sec\times {cm}^{2}}—$ is within the same order of magnitude as the calculation for uncoated membranes –$14.11\;\times 10^{-5}\frac{ml}{sec\times {cm}^{2}}$. Thus, the increased thickness of the membrane does not fully explain the results of the gas transfer measurements through the membrane: results for oxygen saturation did not differ significantly between inlet and outlet of the ENDOXY system. Oxygen consumption of the endothelial cells likewise does not explain the results obtained. Even taking into account a rather high cell density of 0.5 x 10^−6^ / cm^2^, oxygen consumption is lower than the expected gas transfer by two orders of magnitude [32]: $\frac{4.6\;nmol\;O_{2}}{min\times 10^{6}\;cells}\;=\;\frac{10^{4}ml\;O_{2}}{min\times 10^{6}cells}\;=\;\frac{8\times 10^{-7}ml}{sec\times cm²}$. Differences in oxygen saturation between inlet and outlet of the ENDOXY system just reached significance for the uncoated membranes. Thus, we propose that the original ENDOXY system did not allow the detection of gas transfer through endothelialised membranes.

The ENDOXY system was modified to increase its gas transfer capacity. In short, the membrane’s surface area was increased and the chamber’s height reduced, resulting in a more effective gas transfer that was readily measured in whole blood. The gas transfer—$8.46\;\pm 1.62\;\times \;10^{-5}\;\frac{ml}{sec\times {cm}^{2}}—$ was in keeping with the predicted value for endothelialised membranes.

Even though the gas transfer capacity of the system could be increased by these modifications, complete oxygenation of the whole blood could not be achieved. Consequently, different gas-permeable membranes warrant further investigation with regard to their use in a biohybrid lung.

The hydrophobic nature of most gas-permeable membrane surfaces will hamper endothelialisation. Thus, no direct endothelialisation is possible and surface pre-treatment becomes mandatory. The few groups dealing with the endothelialisation of gas permeable membranes for oxygenator used polymethylpenten (PMP) as membrane material and pre-coated the membranes with albumin and heparin or treated the membrane with radio frequency glow discharge (RFGD) and additional gelatine, collagen, and fibronectin coating, respectively [5, 6]. Hess and co-workers [6] could populate the pre-treated PMP with cells, but performed their study under static conditions and did not use whole blood to test the gas transfer. Another approach was tested by Polk *et al*. [5] using rotating PMP bundles coated with ECs.

Rigging these membrane materials that offer higher gas transfer coefficients than the Lumox^™^ membrane for endothelial cell coating will require significant future work.

## Conclusion

We implemented a test system to endothelialise membranes and test the gas transfer properties of these membranes in an oxygenator test loop. Adequate conditioning of the cells by shear stress was observed, and gas transfer could be measured through bare and endothelialised membranes. To achieve adequate oxygenation of whole blood as required in a biohybrid lung, it is necessary to introduce other membranes for comparative analysis to further develop a vital biomimetic composite-membrane with high gas transfer efficiency, high cell retention, and a long persistence.
